# Building a ladder to Hershey Heaven

**DOI:** 10.7554/eLife.15591

**Published:** 2016-03-29

**Authors:** Kai Zinn

**Affiliations:** Division of Biology and Biological Engineering, California Institute of Technology, Pasadena, United Stateszinnk@caltech.edu

**Keywords:** drosophila, resource, protein tagging, live imaging, recombineering, genetics, *D. melanogaster*

## Abstract

A genome-wide resource looks set to turn an experimental ideal into a reality for the *Drosophila* community.

**Related research article** Sarov M, Barz C, Jambor H, Hein MY, Schmied C, Suchold D, Stender B, Janosch S, Vinay Vikas KJ, Krishnan RT, Krishnamoorthy A, Ferreira IRS, Ejsmont RK, Finkl K, Hasse S, Kämpfer P, Plewka N, Vinis E, Schloissnig S, Knust E, Hartenstein V, Mann M, Ramaswami M, VijayRaghavan K, Tomancak P, Schnorrer F. 2016. A genome-wide resource for the analysis of protein localisation in *Drosophila*. *eLife* **5**:e12068. doi: 10.7554/eLife.12068

**Image** Hundreds of fruit fly proteins have been tagged with a fluorescent marker

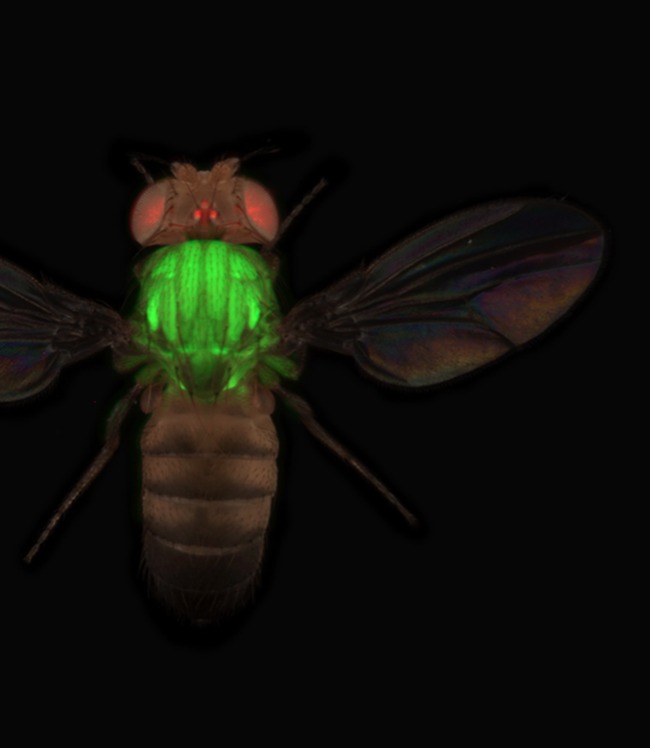

When Alfred Hershey, one of the founders of molecular biology, was asked to describe his idea of scientific happiness, he said that it would be “to have one experiment that works, and keep doing it all the time”. By this he meant that it would be ideal to be able to conduct every experiment using the same tools and methods, and yet always generate new and interesting data (see Creager, 2001). However, molecular geneticists have not yet reached this “Hershey Heaven”. Today, when researchers want to discover more about a protein in an animal – for example, which tissues and cell types express the protein – they usually have to rely on antibodies that bind to the protein of interest. Unfortunately, good antibodies do not exist for most proteins, and it is time-consuming and expensive to generate and characterize new antibodies.

Biologists who work on the model organism *Drosophila melanogaster* have addressed this problem by making “protein traps”. This involves inserting specific sequences into genes in the fruit fly’s genome in order to mark its proteins in a way that makes them easily identifiable without a specific antibody. Some inserted sequences directly encode markers such as fluorescent proteins, while others can be replaced by different marker sequences at a later stage (e.g., Venken et al., 2011; Nagarkar-Jaiswal et al., 2015). Another approach employs transgenic flies that carry an extra functional copy of a gene, with this "third copy" being tagged. Most *Drosophila* genes are relatively compact, which means that they can be contained within DNA fragments that are short enough to be efficiently inserted into the genome.

Now, in eLife, Mihail Sarov, Pavel Tomancak, Frank Schnorrer and colleagues describe a new resource of tagged genes that will be intensively used by all *Drosophila* biologists (Sarov et al., 2016). The researchers – who are based at Max Planck Institutes in Dresden and Martinsried, and other centers in India, the United States and Ireland – generated a library of tagged clones in bacteria for almost 10,000 *Drosophila* genes (which is ~75% of all *Drosophila *genes). Each protein has a multipurpose tag added to its C-terminus, which provides a number of ways to localize or purify a protein of interest, without the need for specific antibodies. The clones are available to the community and can be injected directly into fruit fly embryos to make transgenic lines via the ‘third copy’ strategy. Sarov et al. have already made 880 transgenic lines from the clones, and their data suggest that about two-thirds of the tagged genes will produce functional proteins.

Sarov et al. used the green fluorescent protein in the multipurpose tag to confirm that many of the tagged proteins tended to localize correctly within living cells. They were also able to track protein expression and localization in live animals. Finally, Sarov et al. also demonstrated that tags could be used to purify proteins of interest, along with other components of protein complexes that contain them.

Researchers working with *Drosophila* and other model systems often conduct large genetic screens to identify the genes that control various biological processes. It would be ideal if any new set of genes identified in such a screen could be examined by using a set of transgenic lines and/or clones in which all the genes are tagged in the same way and can be studied using the same tools. The new resource developed by Sarov et al. is a ladder leading toward this experimental heaven, just as Alfred Hershey imagined it.

In the future, researchers will be able to obtain clones for any gene within the new library and make their own transgenic lines. They will then, it is hoped, deposit these new lines in public collections to expand the number available for future study. Finally, Sarov et al. have generated a ‘pre-tagged’ library that is also publicly available. Researchers will now be able to use high-throughput strategies to insert any tags they wish into the genes in this library to make ‘second-generation’ libraries. For example, these could include proteins tagged with different colors so that multiple proteins could be visualized at the same time.

Although this new resource will greatly help work on most *Drosophila* proteins, it comes with some limitations. First, some proteins will be inactivated or destabilized by the addition of tags to the C-terminus. Second, large tags like the ones used in this library may alter the localization or expression of some proteins. Third, some genes encode sets of proteins with different C-termini, and each “isoform” of the protein may have different localizations and/or expression patterns. The present library installs tags on only one of these isoforms. Fourth, ~20% of *Drosophila* genes are too long to be transferred via traditional techniques. All of these problems can be addressed by using CRISPR-based methods to insert tags into any desired position within a gene (e.g., Chen et al., 2015; Gratz et al., 2015). Each gene studied in this manner will represent a separate project. However, if CRISPR-tagged lines for the problem genes also become publicly available, *Drosophila* biologists may eventually be able to study any protein they wish using only publicly available materials. This will greatly speed up research, make it more affordable, and make Hershey Heaven a realistic scenario for the *Drosophila* community.
